# Rescue of Immunotherapy-Refractory Metastatic Merkel Cell Carcinoma With Conventionally Fractionated Radiotherapy and Concurrent Pembrolizumab

**DOI:** 10.3389/fonc.2019.00223

**Published:** 2019-04-05

**Authors:** Brooke C. Bloom, Alexander Augustyn, Todd A. Pezzi, Hari Menon, Lauren L. Mayo, Shalin J. Shah, David L. Schwartz, Steven J. Chmura, Faye M. Johnson, James W. Welsh, Stephen G. Chun

**Affiliations:** ^1^Trinity University, San Antonio, TX, United States; ^2^Department of Radiation Oncology, The University of Texas, MD Anderson Cancer Center, Houston, TX, United States; ^3^Department of Radiation Oncology, University of Tennessee Health Science Center, Memphis, TN, United States; ^4^Department of Radiation and Cellular Oncology, University of Chicago, Chicago, IL, United States; ^5^Division of Cancer Medicine, Department of Thoracic, Head and Neck Medical Oncology, The University of Texas, MD Anderson Cancer Center, Houston, TX, United States; ^6^The University of Texas Graduate School of Biomedical Sciences, Houston, TX, United States

**Keywords:** Merkel cell carcinoma, radiation, abscopal, PD-1, PD-L1, immunotherapy, checkpoint

## Abstract

Merkel cell carcinoma has historically had dismal prognosis with limited cytotoxic chemotherapy options that provide durable control of metastatic disease. The advent of anti-programmed death protein (anti-PD1)/anti-programmed death-ligand 1 (anti-PD-L1) directed immunotherapy has shown initial promise in Merkel cell carcinoma and radiation might augment immune responses. We present a case report of a 70-year-old male who underwent resection of Merkel cell carcinoma of the right thigh with a close margin and positive right inguinal involvement. Due to high-risk features, the patient was treated with adjuvant radiation to the right groin and with systemic carboplatin/etoposide, but developed local failure requiring salvage surgical resection. The patient then developed metastatic disease with biopsy proven retroperitoneal involvement refractory to doxorubicin/cyclophosphamide chemotherapy. The patient was then transitioned to single-agent pembrolizumab with a partial response for 10 months until developing progressive disease involving the left inguinal and left external iliac nodal regions. The progressive left inguinal/pelvic disease was treated with conventionally fractionated intensity modulated radiation therapy to a dose of 45 Gy delivered in 25 fractions. Following radiation therapy, the patient had complete response of all sites of disease throughout the body on imaging by RECIST criteria including retroperitoneal and mediastinal disease outside the radiation field. At 20 months post-radiation, the patient remains on pembrolizumab without evidence of disease on imaging. Herein, we present a case of durable response of metastatic Merkel cell carcinoma treated with concurrent radiation and pembrolizumab, providing evidence that radiation might improve systemic responses to anti-PD1/PD-L1 directed immune therapy. Ongoing prospective trials evaluating the utility of radiation in conjunction with immunotherapy for Merkel cell carcinoma are anticipated to provide clarity on the frequency and durability of abscopal responses when radiation is combined with immune checkpoint inhibitors.

## Background

Merkel cell carcinoma is an aggressive cutaneous neuroendocrine malignancy with roughly 1,600 cases diagnosed annually in the United States and increasing incidence (1, 2). For metastatic Merkel cell carcinoma, cytotoxic chemotherapy and radiation can provide palliation, but responses to cytotoxic chemotherapy have limited durability and long-term survival outcomes have historically been dismal (3, 4). As locoregional failure and metastatic dissemination frequently occur with Merkel cell carcinoma, there is great interest in pursuing novel systemic treatment strategies such as anti-programmed cell death protein 1 (PD1)/anti-programmed death-ligand 1 (PD-L1) directed immunotherapy to improve outcomes for this malignancy (5).

Anti-PD1 and anti-PD-L1 directed therapies are immune checkpoint inhibitors that have shown initial promise in the treatment of Merkel cell carcinoma. As 80–90% of Merkel cell carcinomas are associated with the Merkel polyomavirus and expression of viral T-cell antigens (6, 7), immune perturbation has long been hypothesized to play a central role in Merkel carcinogenesis. Furthermore, chronic immunosuppression is also linked to the development of Merkel cell carcinoma (8), further suggesting that immune dysregulation is a hallmark of this malignancy. About 55% of Merkel cell carcinomas overexpress PD-L1 (9), and a Phase II clinical trial using pembrolizumab showed a 56% response rate in patients who had not previously received systemic therapy (10). When used as second-line therapy, the anti-PD-L1 antibody avelumab showed a response rate of 33% (11). These results led to Federal Drug Administration (FDA) approval of avelumab for metastatic Merkel cell carcinoma.

There is also rationale to use radiation therapy in conjunction with checkpoint inhibitors to augment systemic immune responses. The often purported abscopal effect defined as tumor responses outside radiation fields/volumes may occur through multiple mechanisms such as increased tumor antigen exposure and presentation, improved T-cell function, and in this case potentially (4–14) direct tumor debulking by radiation decreasing CD8 T-cell exclusion (15–17).

Similar to other neuroendocrine malignancies, Merkel cell carcinoma is known to be a radiation responsive malignancy (12), and pre-clinical tumor models have shown that radiation might increase tumor antigen presentation and affect T-cell signaling (13–15). In mouse tumor models, radiation combined with checkpoint inhibition has been shown to improve tumor responses. Furthermore, the abscopal response has been reported in patients with metastatic Merkel cell carcinoma treated with radiation therapy to a dose of 8 Gy in a single fraction with external beam radiation (13), and high dose rate (HDR) brachytherapy to a dose of 12 Gy delivered in 2 fractions (16).

We present a case report of a patient with metastatic Merkel cell carcinoma on anti-PD1 therapy, treated with conventionally fractionated radiation therapy for progression in the left inguinal nodal region resulting in durable abscopal response at other metastatic sites outside the radiation field. This case provides additional rationale to explore the effect of radiation therapy for metastatic Merkel cell carcinoma being treated with checkpoint inhibitors.

## Case Report

**Figure 1:**
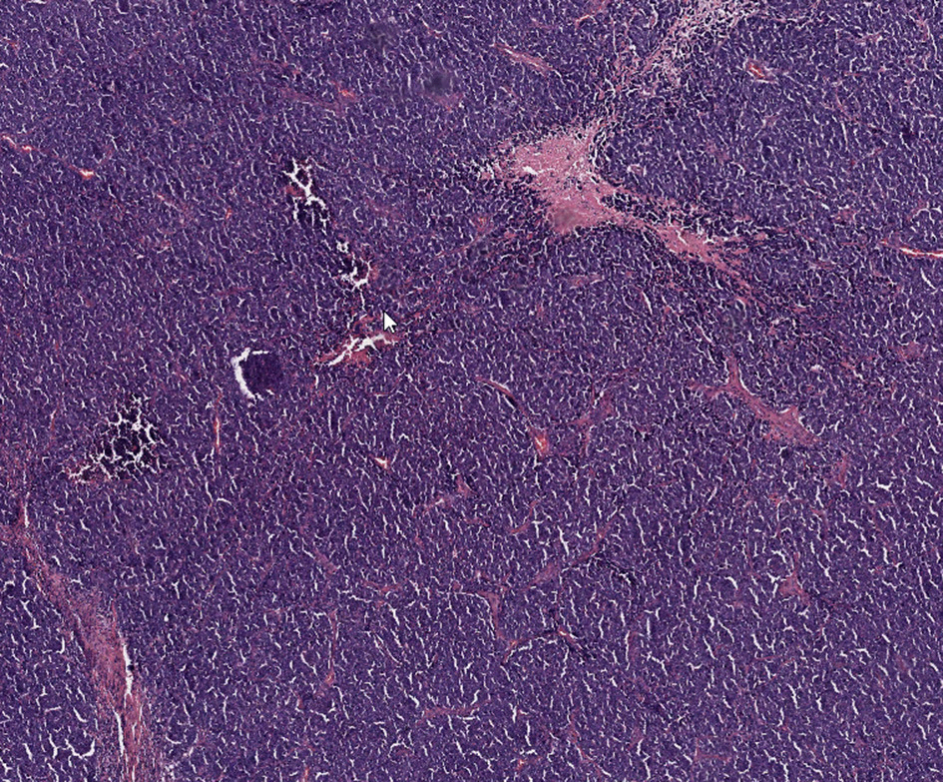
Representative histologic image of patient's Merkel cell carcinoma with hematoxylin and eosin staining.

**Figure 2:**
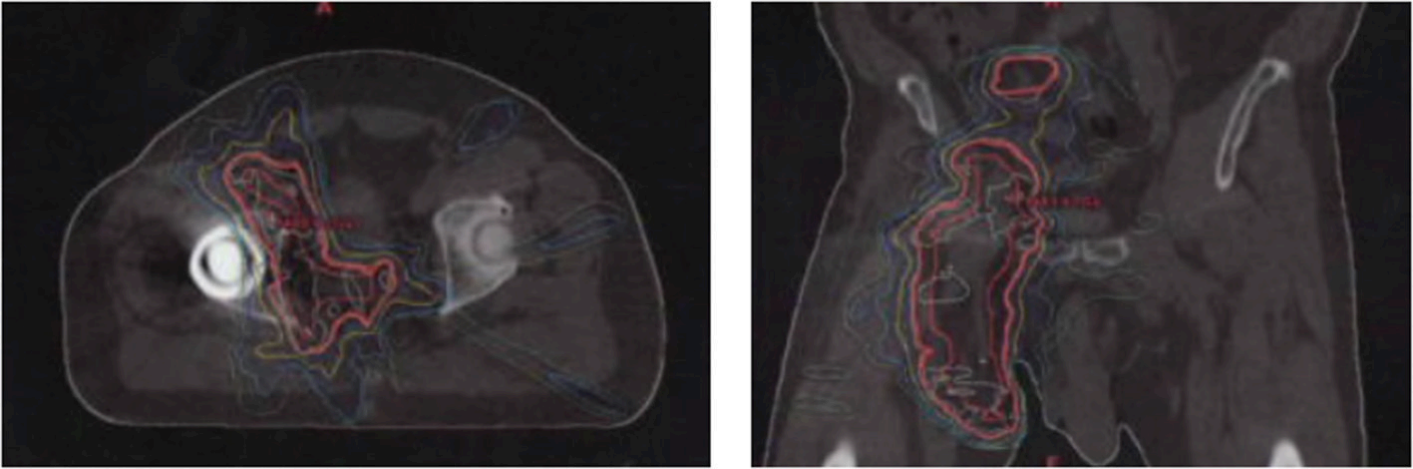
Adjuvant radiation therapy plan of right thigh and groin showing axial and coronal representation.

A 70-year-old male was diagnosed with Merkel Cell carcinoma of the right thigh (Figure 1). The patient underwent surgical resection with close (<1 mm) surgical margins in 2012. Surgical pathology showed the tumor to be CD56, synaptophysin, and pancytokeratin positive by immunohistochemistry, with involvement of a single right inguinal lymph node. Due to close surgical margins and inguinal nodal involvement, the patient received adjuvant intensity modulated radiation therapy (IMRT) to the right thigh and right pelvic and inguinal nodal region to a dose of 50 Gy delivered in 25 fractions (Figure 2), as well as consolidative carboplatin and etoposide post-operatively. The patient then developed a radiation in-field recurrence involving the right external iliac and inguinal nodal region in 2015. Salvage right pelvic nodal dissection was performed where 15 nodes were removed with four harboring metastatic Merkel cell carcinoma.

**Figure 3:**
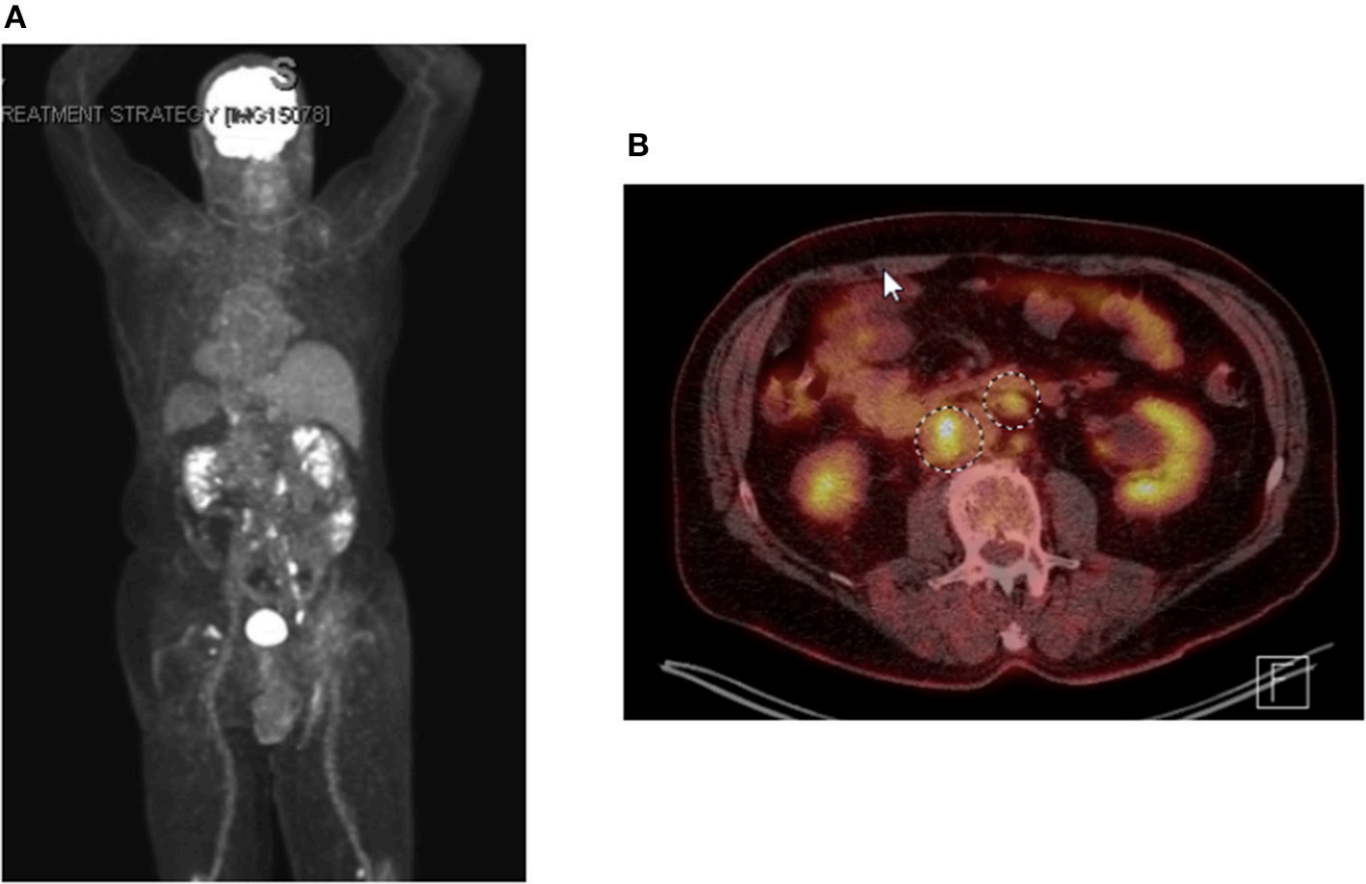
PET-imaging demonstrating metastatic dissemination of Merkel cell carcinoma. **(A)** whole body positronic imaging showing bilateral pelvic and retroperitoneal SUV avidity consistent with metastatic disease. **(B)** representative fused PET CT-scan axial imaging showing SUV avid retroperitoneal adenopathy.

**Figure 4:**
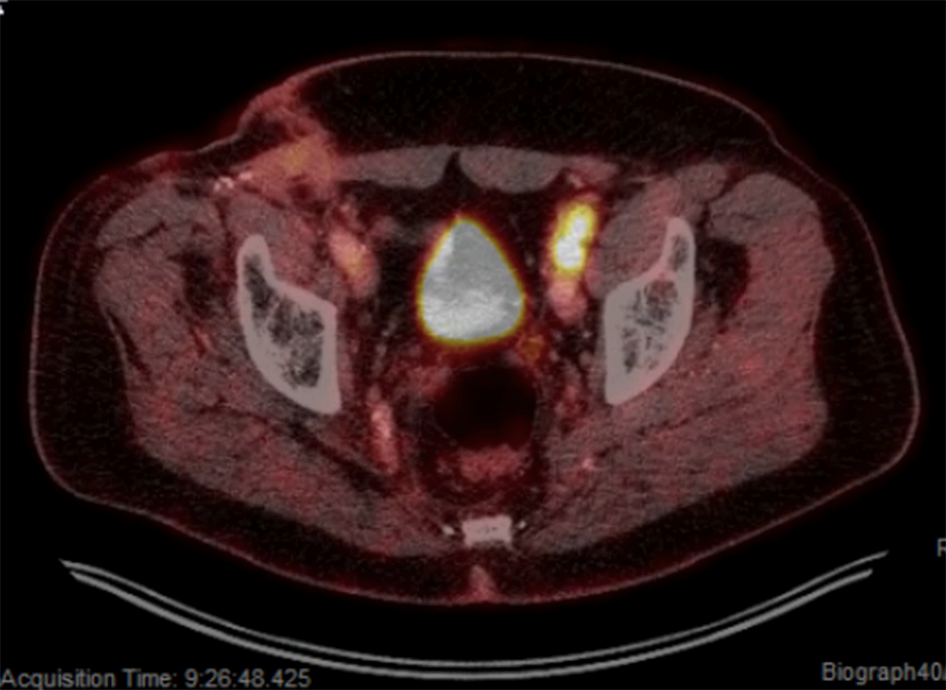
Isolated left pelvic nodal progression on pembrolizumab. PET-scan showing SUV avid progression involving the left inguinal and left external iliac regions.

**Figure 5:**
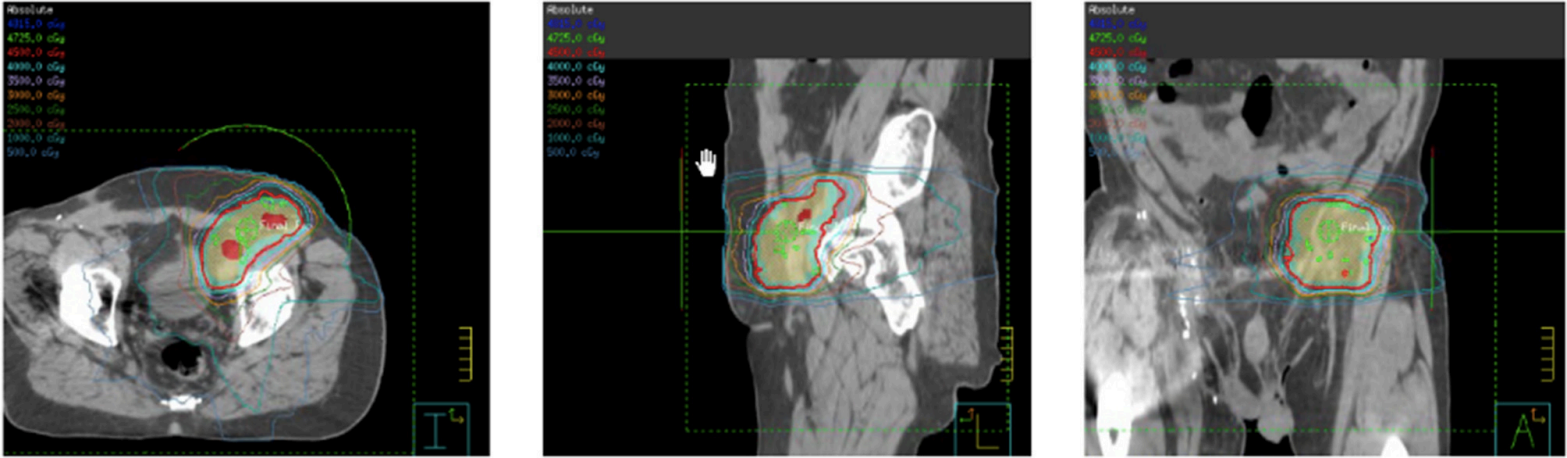
Intensity modulated radiation therapy (IMRT) plan for left pelvic nodal progression to a prescription dose of 45 Gy delivered in 25 fractions with concurrent pembrolizumab.

Shortly after locoregional recurrence in the right pelvic nodal region, the patient developed metastatic disease involving bilateral pelvic nodes and the retroperitoneum on PET-scan (Figure 3). Biopsy of the SUV-avid retroperitoneal lymphadenopathy confirmed metastatic Merkel cell carcinoma. The patient was treated initially with doxorubicin and cyclophosphamide and due to progressive disease was transitioned to single-agent pembrolizumab (200 mg IV every 3 weeks). After 5 months of therapy (7 cycles of pembrolizumab) the patient had a complete metabolic response with only sub-centimeter lymph nodes visible on CT. After 10 months of pembrolizumab (15 cycles), the patient had an isolated left inguinal and external iliac nodal progression (Figure 4). For this reason, the patient was treated to the involved left inguinal and left external iliac nodes with IMRT to a dose of 45 Gy delivered in 25 fractions with concurrent pembrolizumab (Figure 5), without any acute radiation side effects. Thereafter, the patient had complete imaging response of the left pelvic nodes as well as all other sites of metastatic disease outside of the radiation field at 3 months post-radiation by PET-response criteria in solid tumors (PERCIST) criteria (17). At the present time (20 months post-radiation), the patient has received 39 cycles of pembrolizumab without evidence of cancer on serial imaging with mild rash as the only immune-related side effect.

## Discussion

Although immune checkpoint blockade has emerged as an important treatment option for metastatic Merkel cell carcinoma, not all patients experience durable disease control. We report a case of metastatic Merkel cell carcinoma with progressive inguinal nodal disease by RECIST and PERCIST criteria on pembrolizumab treated with radiation therapy with durable disease control locally and systemically. While the first reported case of an abscopal response in metastatic Merkel cell carcinoma was observed in 2011 in a patient treated with radiation therapy alone, this case builds upon evidence from pre-clinical and clinical observations suggesting that using radiation therapy while targeting the PD-1/PD-L1 axis has the potential to improve systemic responses (15, 18). This is unique as the patient was progressing on PD-1 therapy and the radiation therapy was the only treatment given that resulted in the conversion from a RECIST progressive disease to a complete response.

This case report also provides further rationale for the ongoing clinical trials including NCT03071406 (A Randomized Study of Nivolumab and Ipilimumab with or without stereotactic body radiation therapy for Metastatic Merkel Cell Carcinoma), as well as the Alliance Oncology Cooperative Group trial (A091605) where patients with metastatic Merkel cell carcinoma being treated with pembrolizumab are randomized to receive radiation therapy. The Alliance trial is unique as the primary endpoint is progression-free survival in *non-irradiated* lesions in those receiving either SBRT and pembrolizumab or pembrolizumab alone. This case observation would suggest a benefit exists with the addition of radiation therapy in regards to this particular endpoint of interest.

Another unique aspect of this case was the use of fractionated radiation therapy in conjunction with anti-PD1 directed therapy. While hypofractionated radiation has previously been reported to induce the abscopal response in Merkel cell carcinoma (13, 16), our reported case suggests that conventionally fractionated radiotherapy may be capable of achieving similar responses. As neuroendocrine malignancies have traditionally been reported to respond robustly to conventionally fractionated radiation (1.8–3 Gy), our case suggests that standard fraction sizes may be sufficient to induce systemic immune responses. Indeed, preclinical data exists suggesting that high single fraction doses attenuate radiotherapy-induced immunogenicity by promoting exonuclease function and degrading cytosolic DNA, which is an essential stimulant for the priming of CD8^+^ T cells. Meanwhile, fractionated doses resulted in increased type I interferon production and subsequent CD8^+^ T cell activation (19). Furthermore, in a comprehensive review of reported abscopal responses, the majority were elicited by conventionally fractionated radiation (20). The optimal dose fractionation needed to induce systemic immune responses remains an area of open debate. The biologic effects of conventionally fractionated radiation therapy and hypofractionated radiation vary significantly between different tumor histologies and normal tissue stroma type. For this reason, a therapeutic strategy tailored to both tumor histology and anatomic site will likely be important to optimize the therapeutic window when combining radiation with checkpoint inhibitors.

In summary, we have presented a case of metastatic Merkel cell carcinoma with progression on pembrolizumab for which conventionally fractionated radiation resulted in a durable systemic abscopal response. This case builds upon growing literature validating the occurrence of the abscopal effect when using radiation therapy in Merkel cell carcinoma. As such, the utilization of combined modality strategies combining radiation and checkpoint inhibitors should be explored enthusiastically in Merkel cell carcinoma.

## Author Contributions

All authors listed have made a substantial, direct and intellectual contribution to the work, and approved it for publication.

### Conflict of Interest Statement

The authors declare that the research was conducted in the absence of any commercial or financial relationships that could be construed as a potential conflict of interest.
